# The 2009 US Federal Cigarette Tax Increase and Quitline Utilization in 16 States

**DOI:** 10.1155/2012/314740

**Published:** 2012-05-08

**Authors:** Terry Bush, Susan Zbikowski, Lisa Mahoney, Mona Deprey, Paul D. Mowery, Brooke Magnusson

**Affiliations:** ^1^Alere Wellbeing (Formerly Free & Clear, Inc.), 999 Third Avenue Suite 2100, Seattle, WA 98104-1139, USA; ^2^Biostatistics, Inc., 228 E Wesley Road Ne, Atlanta, GA 30305-3710, USA

## Abstract

*Background*. On April 1, 2009, the federal cigarette excise tax increased from 39 cents to $1.01 per pack. *Methods.* This study describes call volumes to 16 state quitlines, characteristics of callers and cessation outcomes before and after the tax. *Results*. Calls to the quitlines increased by 23.5% in 2009 and more whites, smokers ≥ 25 years of age, smokers of shorter duration, those with less education, and those who live with smokers called after (versus before) the tax. Quit rates at 7 months did not differ before versus after tax. *Conclusions*. Descriptive analyses revealed that the federal excise tax on cigarettes was associated with increased calls to quitlines but multivariate analyses revealed no difference in quit rates. However, more callers at the same quit rate indicates an increase in total number of successful quitters. If revenue obtained from increased taxation on cigarettes is put into cessation treatment, then it is likely future excise taxes would have an even greater effect.

## 1. Introduction

On February 4, 2009, a 62-cent increase in the federal cigarette tax was enacted, along with increases in other tobacco taxes, to fund expansion of the State Children's Health Insurance Program (SCHIP) [1]. The federal cigarette tax increased to $1.01 per pack on April 1, 2009. Increasing the price of tobacco through excise taxes is an effective way to encourage quit attempts and thus to decrease the prevalence of smoking [2, 3]. It is estimated that a 10% increase in cigarette prices leads to a 4% decrease in cigarette consumption in high-income countries and about 8% in low-to-middle income countries [2, 4]. A 70% increase in current tobacco prices could prevent 25% of all smoking-related deaths globally [4] and higher taxes have a greater impact on the young and low income smokers by deterring smoking initiation and encouraging smokers to quit [2]. Telephone quitlines are an effective population-based form of smoking cessation treatment and their utilization has been shown to be responsive to tobacco control policies [5–9]. Therefore, the study aims were to (1) describe call volumes to 16 state quitlines before and after the tax increase; (2) examine the characteristics of tobacco users who enrolled with quitlines before and after the tax increase and (3) examine the outcomes (quit rates) of tobacco users who enrolled with state quitlines before and after the tax increase. Analyses were conducted to determine whether implementation of the federal tax on April 1, 2009 coincided with (1) increased calls to state quitlines; (2) increased calls from people with low education levels; (3) increased quit rates (the higher cigarette prices may motivate those attempting to quit to remain quit). It was expected that the increase in calls may begin prior to April 1, 2009 and as early as February 2009 as people become aware of the passage of the federal tax increase and in response to preemptive cigarette price increases instituted by the tobacco industry in December 2008 and March 2009 [10, 11].

## 2. Methods

Two different data sources were used: one is based on administrative data collected from all callers and the other is a seven-month follow-up interview with a random sample of quitline participants. Administrative data comes from the Free & Clear database for state tobacco quitlines that tracks call volumes, completed counseling calls and caller characteristics obtained during registration with the quitline. This data comes from 16 of the 17 state quitlines operated by Free & Clear, Inc., at the time of this study. The one state that was not included in the analysis had incomplete data for the time period before the tax increase. Participating states include Alaska, Connecticut, Georgia, Hawaii, Indiana, Maine, Maryland, Missouri, North Carolina, Oklahoma, Oregon, South Carolina, Utah, Virginia, Washington and Wisconsin, whose smokers represented 24% of smokers in the United States in 2009 [12]. Data from the seven-month followup comes from four state quitlines and is based on random samples of quitline participants in each state timed to occur seven months from enrollment with the quitline. All 16 states agreed to participate in this study and to contribute their data to the pooled dataset. The 16 state quitlines represent different geographic regions and states with varying state laws (e.g., tobacco control programs with varying resources and programmatic activities). As well, they have a variety of cessation services such as offering free nicotine replacement therapy (NRT) through their quitline during the study period. Since states regularly change their services, we chose to portray a snapshot of offerings during the study period (Table 1). All quitlines provided mailed support materials (Quit Guides), a single reactive (inbound) counseling call to all tobacco users, and three or four additional outbound calls to select groups (e.g., those ready to quit within 30-days). Some state quitlines refer insured tobacco users to cessation benefits offered through their health plan or employer. All but four states offered at least some free NRT (patch or gum) depending on the state-approved eligibility criteria (e.g., insurance status). All of the participating states used the same data collection methods and a common questionnaire [13] to collect demographic and tobacco use data at intake and follow-up thus enhancing data comparability across state quitlines and across study years.

## 3. Measures

### 3.1. Total Calls to State Quitlines

Analyses examined both pooled monthly call volume (total calls to quitlines) and pooled daily call volume. State-level data was not examined as this was a descriptive study to assess whether a volume change would be observed in aggregate data from callers to the quitline in 16 states. However, in statistical models of outcomes, “state” was included as a fixed effect to account for unmeasured variability within and between states. Monthly data was examined from December 2008 through August 2009 and from a similar time period the year before for comparison (December 2007 through August 2008) in order to show call volume prior to the tax increase (December 2008, January 2009, February 2009, March 2009), during the months that the tobacco industry increased prices in anticipation of the tax increase (December 2008 through March 2009), during the month the tax increase was passed (February 2009), and after the tax increase took effect (April 2009, May 2009 anticipated to have heavy call volumes). Daily call volume was then examined directly before and after the April 1, 2009 tax increase to determine when the calls began to increase in anticipation of the tax increase and how soon the calls returned to previous levels. The time period selected for this analysis was March-April 2009 (March-April 2008 was also examined for comparative purposes).

### 3.2. Caller Characteristics

A comprehensive set of variables collected when a person enrolls with a state quitline was used to describe caller characteristics. Variables included participant demographics (age, gender, race/ethnicity, insurance status, educational level), current tobacco use (tobacco type, amount used), duration of smoking, time to first cigarette upon waking, living or working with smokers, and how they heard about the quitline. Chronic disease status was assessed by asking: “have you been diagnosed with any of the following conditions; asthma, chronic obstructive pulmonary disease or emphysema, coronary artery disease or heart disease or diabetes?” Responses were captured with a “yes” or “no” to each chronic disease.

### 3.3. Seven-Month Quit Rates

Data from the seven-month followup survey was obtained for persons who enrolled from March 2009–May 2009 (and for comparison March 2008–May 2008). This time period was selected for comparisons of demographics and quit rates because this was the period in which the impact of the tax would most likely be observed. The four states with available data (i.e., some did not conduct the seven-month survey during these time periods and others used another organization to conduct the seven-month survey and their data was unavailable) used similar survey sampling protocols and similar questionnaires. Information collected at the seven-month follow-up included use of medications since enrolling with the quitline and current smoking status. Successful cessation was defined as seven-day and 30-day abstinence by asking participants: “when was the last time you smoked a cigarette, even a puff?” Our questionnaire included the standard battery of questions used in the Minimum Data Set (MDS) instrument recommended by the North American Quitline Consortium (NAQC) [13]. The seven-month response rate was 39.3%.

## 4. Analyses

Data across all states was pooled and presented in aggregate form in graphs and tables. First, the total number of monthly calls to the 16 quitlines was collected and presented in a figure as well as the number of tobacco users who received one or more counseling calls from December 2007 through August 2008 and December 2008 through August 2009. Rao-Scott Chi-square and *t*-test statistics were used to compare characteristics of callers during the time of the 2009 tax increase and for the same months in the prior year and included state as a fixed variable to account for the variability in services provided across quitlines. A *P* value of 0.01 was used as a cut-off value for the hypotheses tests because of the large sample size. For the four states with data from the seven-month follow-up, multivariate logistic regression analyses were used to examine tobacco abstinence outcomes (7-day point prevalence and 30-day point prevalence) comparing callers during the time of the 2009 tax increase to callers during the same time period in the prior year. Again, “state” was included as a fixed variable, as well as case-mix covariates that differed before versus after the tax increase (age, race, education, chronic conditions, how they heard about the quitline, and amount smoked at intake), and gender because it is associated with cessation outcomes [14, 15]. For those who enrolled with the multicall program, utilization of services (number of counseling calls completed) and quit outcomes was also assessed in multivariate and logistic regression analyses. Outcomes were reported in two ways: first among those who completed the survey (respondent analysis) and second using the “intent-to-treat” (ITT) analysis whereby persons with missing outcomes data are assumed to be smoking.

The logistic model was

*Logit(probability of abstinence (yes/no)) = overall mean+time indicator variable+individual covariates + state + callprogram + howheard, where time indicator variable = before or after (0 or 1), callprogram = one versus multicall program and howheard = how participant heard about the Quitline*

## 5. Results

**Figure 1:**
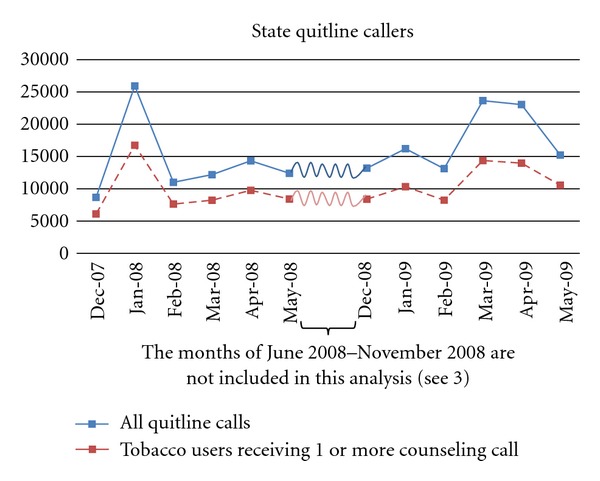
**Monthly number of calls and number who received counseling from 16 state quitlines, December 2007 through May 2009** ((1) See Table 1 for a description of services within the participating states. (2) All quitline calls (top line) include proxy callers, providers, general public, “hang ups,” tobacco users wanting materials only, seeking treatment, or those enrolled who call back to speak with coach. Tobacco users (bottom line) represent those enrolled in the quitline who completed at least one counseling call. (3) The spike in January 2008 is primarily due to a cigarette tax increase in one large state and associated promotional activities. Call volumes tapered after May 2008 and May 2009, thus data from May 2008–November 2008 and after May 2009 are not included in the graph).

**Figure 2:**
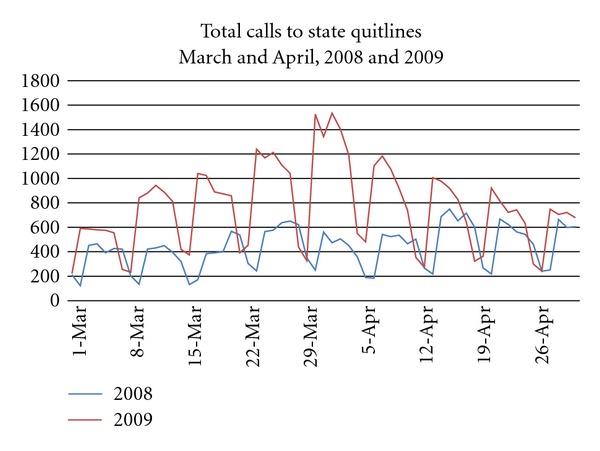
Daily number of calls to 16 state quitlines. All listed dates are Mondays.

Figure 1 presents the number of calls to the 16 quitlines over time and shows the spike in calls during March-April 2009. Overall, there was a 23.5% increase in total call volume when comparing December 2007–May 2008 (84,541 calls) to December 2008–May 2009 (104,452 calls). In 2009, calls increased beginning in March and began to taper off in May (a 59.1% increase in call volumes comparing March 2008–May 2008 (38,919 calls) to March 2009–May 2009 (61,935 calls)). Comparing each month in 2008-2009 with the corresponding month from the prior year, increases in call volume were observed in December 2008 and February through May 2009, with the largest percent increase (94.1%) occurring in March 2009. Increases during March and April 2009 occurred both in total call volume (calls from tobacco users, friends, family, health care professionals, and the general public seeking information), as well as in the number of tobacco users per month who received at least one counseling call. Data for June, July, and August are not shown in Figure 1 since the tax effect on call volumes had returned to the before tax levels in May. Note that the observed increase in quitline calls (and enrollments) around January 1 for both time periods was expected and is often attributed to New Years' resolutions. Some states also plan promotional events to coincide with this seasonal effect. This was the case in January 2008 whereby the spike in calls corresponds to promotional activities of one large state quitline [16]. In post hoc analyses, omitting this state from the sample resulted in a similar pattern (although a lower number of calls) as that shown in Figure 1.  Figure 2 shows a more detailed analysis of call volumes around the April 2009 tax increase; the daily call volumes from March through April 2009 compared to daily call volumes from March through April 2008. Daily call volume was higher in 2009 than 2008, particularly from March 8 through April 26. The dips in the figure represent weekends when call volumes are traditionally low.

Table 2 shows results of the comparisons of demographic and other characteristics between tobacco users who enrolled with the quitline before and after the announcement and implementation of the April 2009 federal tax increase. The time periods for this analysis were March 1, 2008 through May 31, 2008 versus March 1, 2009 through May 31, 2009 (the window of time showing the peak activity in call volumes). Results reveal differences in callers between the two time periods. In the after tax period, although the mean age of callers was slightly younger (41.9 versus 41.2), fewer callers were aged 18–24 years (11.5% after tax versus 13.6% before tax). More callers in 2009 (compared with the prior year) were white, had less than a high school education, were more likely to live with a smoker, had shorter durations of cigarettes smoking, and were more likely to report hearing about the quitline from family or friends or their health care provider, rather than from the media. Although fewer callers enrolled in the multicall program (4-5 counseling calls) after tax, they completed slightly more counseling sessions compared with those who enrolled for the multiple calls before tax (1.9 versus 2.2, respectively, *P* < 0.0001). Although there were differences in the prevalence of chronic disease (COPD and CAD) and use of other tobacco products when comparing callers after the tax increase to those before the tax increase, these differences between the callers in these two time periods were small.

Table 3 shows results of analyses of seven-month outcomes data and suggests that participant quit rates did not differ significantly before versus after the tax. These results held for unadjusted and adjusted analyses of seven-day and 30-day respondent and intent-to-treat analyses. For example, seven-day respondent quit rates were 30.7% before and 28.7% after the tax (O.R. = 0.95, 95% C.I. = 0.63, 1.45). Analyses of the subgroup that participated in the multicall program showed a similar lack of change in smoking status after tax compared with a similar period before the tax.

## 6. Discussion

This study's results are consistent with prior research showing that implementing an increase in excise taxes on tobacco will drive calls to the state tobacco control programs' free quitline services [3, 17]. Harwell et al. reported an increase in call volumes to the Montana quitline following an increase in the state's cigarette taxes. They also reported that the tax attracted younger smokers to call the quitline, as well as more female and white smokers and heavier smokers [3]. In the current study, although smokers who called the quitline around the time of the federal tax increase were more likely to be white, no significant differences were found for gender or amount smoked. However, fewer young tobacco users (age 18–24) and fewer smokers with smoking durations of ≥20 years called around the time of the tax increase. Because tax increases tend to decrease the prevalence of smoking among younger persons and persons with lower incomes more than older persons and those with higher incomes [2], it was expected that differences would emerge in these demographic characteristics in callers during the time of the tax increase compared to those who called the year before. Although persons with lower education levels were more likely to call after the tax increase, young adults were slightly less likely to call. However, for all characteristics, the magnitude of the differences before and after the tax increase was small.

Observed changes in who called the quitline around the time of the tax increase versus the year before could be due to multiple factors such as state quitline promotional efforts that were timed to correspond to the tax increase as well as the local increases in actual cigarette prices themselves. This study is descriptive in nature and did not address the myriad of changes in tobacco control policies and interventions that may have occurred at the state and local levels during this time period and how those changes would have influenced both the number of calls to the quitline and demographic and other characteristics of quitline callers. For example, in addition to the federal excise tax increase in April 2009, 13 states in this study increased their cigarette excise taxes between November 2008 and November 2009. More research would be needed to estimate the effect of the federal tax increase apart from these and other changes that were occurring at the state and local levels.

Interestingly, in terms of caller characteristics, the variable that changed the most among callers around the time of the federal tax increase compared to the year before was how the caller heard about the quitline. Callers after the tax increase were more likely to report that friends and family told them about the quitline than those who called before the tax increase. Future research could explore how cigarette tax increases influence friends' and families' interest in encouraging and assisting smokers with cessation. Additional research is also needed to determine the synergistic effects on call volumes and treatment outcomes of state promotional events that may have coincided with implementation of the tax increase.

The lack of higher quit rates after the tax is not surprising since the quitlines did not provide additional counseling or other services for tobacco users after the tax and in fact may have reduced the availability of more intensive cessation treatments (see Table 1) [18]. Although the quit rates were similar before and after the federal tax increase, the number of tobacco users who enrolled in the quitlines was larger after the tax increase. Therefore, in terms of absolute numbers, more persons successfully quit after the tax increase. In these 16 states, of the 19,911 additional tobacco users who called during the time of the tax an additional 5,714 would quit smoking (19,911 more callers after tax ∗ 28.7% quit rate). However, it is important to remember that only 1%–5% of smokers in the United States call quitlines each year and tobacco users often quit without the use of cessation services or medications [19]. Increasing the price of cigarettes is associated with increase quitting (1) and future research could examine the effects of the increase in the federal excise tax on more general population-based measures of cessation.

## 7. Limitations

Results of this study must take into consideration a number of potential limitations. One limitation is that the number and types of callers to quitlines vary within and between states over time and are a function of promotional events (e.g., offering free NRT) and eligibility criteria (e.g., NRT for uninsured only) that were not examined in this analysis. Note that the services provided did not change before/after in the four states with 7-month data. Furthermore, since the primary intention of this paper is to describe the populations using the quitlines around the tax increase, it is likely that the data accurately portrays the types of callers who were calling around that time. Although analyses of individual states' promotional activities or other tobacco control initiatives were not conducted, “state” was included in the statistical models to control for such variability. Note that the pattern of calls was similar in graphs with and without one outlier state that had paired the normal January increase in calls with a state tax increase and promotional activities around the quitline. Future studies should consider including a more detailed analysis of promotional efforts as well as state-specific tax increases. Unfortunately, there is no data source currently available that tracks the amount, content, and timing of state antitobacco promotional efforts [20, 21]. Another consideration is that data were missing for both time periods for over 10% of enrolled callers at intake for four variables (education, presence of other household smokers, years of tobacco use, and whether they were mailed NRT by the quitline). This is a reasonable amount of missing responses for these specific measures obtained during quitline enrollment. However, results for those variables may have been influenced by this nonresponse although it is difficult to predict the magnitude and direction of how the nonresponse would affect the relationship to the tax increase. In addition, only four states had seven-month quit rate data that spanned the study period; therefore, these quit rate results might not generalize to other quitlines. Analyses compared cessation rates among those who enrolled around the time of the tax increase compared to persons who enrolled during the same months during the prior year but there were no questions to determine if success was due to the individual's interaction with the quitline. Also, seven-month survey response rates tend to be fairly low. Low response rates are a common finding in phone-based follow-up surveys with individuals seeking treatments. Response rates in the 30–40% range are reasonable and consistent with other studies (NAQC 2009). Although analyses controlled for response rates by reporting the intent to treat quit rates, the assumption that nonrespondents are continued smokers has been challenged as being too conservative [22]. Cessation rates may have differed significantly between time periods if higher response rates had been obtained. The true quit rates will lie somewhere between the responder and intent-to-treat results. Because of the above limitations, conclusions about changes in quit rates among quitline callers after the federal tax increase should be interpreted with caution.

## 8. Conclusions

This study provides important data relevant to public health policy on tobacco control. Evidence-based cessation services combined with tax and price increases, smoke-free laws, antitobacco advertising, and bans on tobacco advertising and promotion increase cessation and decrease tobacco use prevalence [2]. Frieden and colleagues found that intensive tobacco control measures decreased the prevalence of smoking by 11% among New York City adults from 2002 to 2003 and estimated that 59% of that reduction in smoking was due to price increases [23]. Further, the interactive effects of multiple policies are more effective and have a greater public health impact when combined with other evidence-based components of tobacco control programs [24]. States must ensure that consumers have access to effective services (including quitlines) [25]. However, in a recent survey of quitline service providers, 89% reported that reduced funding had a direct effect on provision of services (e.g., limiting eligibility for services, reducing the number of counseling sessions, or eliminating provision of NRT) [18]. This is unfortunate since offering free NRT through the Quitline can increase calls and increase cessation [5, 7, 9]. In the current study, variability in the type and intensity of cessation services (e.g., number of counseling sessions, amount of NRT offered) provided by each state over the two time periods may have been due to budgetary constraints [6]. Through *Best Practices for Comprehensive Tobacco Control Programs,* CDC recommends funding levels for comprehensive tobacco control programs, including effective interventions such as quitlines [26]. If all states met CDC's recommended annual levels of funding for tobacco control programs ($9–$18 per capita), in five years, an estimated five million fewer persons would smoke and hundreds of thousands of premature tobacco-related deaths could be prevented [27].

## Figures and Tables

**Table 1:** Benefits and services offered by the 16 state quitlines participating in the study before and after the federal excise tax increase.^1, 2^

State quitline	Mailed materials		1 single, reactive		Single call + 3 additional calls^3^		Single call + 4 additional calls^3^		Free NRT (2 weeks)^3^		Free NRT (4 weeks)^3^		Free NRT (8 weeks)^3^		Free NRT (12 weeks)^3^	
	before	after	before	after	before	after	before	after	before	after	before	after	before	after	before	after
State 1	Y	Y	Y	Y	—	—	Y	Y	Y	—	—	—	—	Y	—	—
State 2	Y	Y	Y	Y	—	—	Y	Y	—	—	—	—	Y	—	—	—
State 3	Y	Y	Y	Y	—	—	Y	Y	—	—	—	—	Y oth	—	—	—
State 4	Y	Y	Y	Y	Y	Y	—	—	Y	—	—	—	—	—	—	—
State 5	Y	Y	Y	Y	Y	Y	—	—	Y	Y	—	—	—	—	—	—
State 6^4^	Y	Y	Y	Y	Y	Y	**—**	**—**	**—**	**—**	**—**	**—**	**—**	**—**	**—**	**—**
State 7	Y	Y	Y	Y	Y	Y	—	—	—	—	—	—	Y	—	Y	Y
State 8	Y	Y	Y	Y	Y	Y	—	—	—	—	—	Y oth	—	—	—	—
State 9^4^	Y	Y	Y	Y	Y	Y	**—**	**—**	**—**	**—**	**—**	**—**	**—**	**—**	**—**	**—**
State 10	Y	Y	Y	Y	—	—	Y	Y	Y ins	Y med	—	—	Y unins	Y unins	—	—
State 11	Y	Y	Y	Y	Y	Y	—	—	Y ins	—	—	—	Y unins	—	—	—
State 12^4^	Y	Y	Y	Y	—	—	Y	Y	**—**	**—**	**—**	**—**	**—**	**—**	**—**	**—**
State 13	Y	Y	Y	Y	—	—	Y	Y	—	—	Y ins	Y ins	Y unins	Y unins	—	—
State 14^4^	Y	Y	Y	Y	Y	Y	**—**	**—**	**—**	**—**	**—**	**—**	**—**	**—**	**—**	**—**
State 15	Y	Y	Y	Y	—	—	Y	—	Y ins	—	—	—	Y unins	—	—	—
State 16	Y	Y	Y	Y	—	—	Y	Y	—	—	—	Y oth	—	—	—	—

^1^Restrictions apply: Y ins = NRT provided to insured callers only; Y unins = NRT provided to uninsured callers only; Y med = NRT provided to Medicaid callers only; oth = other restrictions apply; Y = no restrictions. ^2^Before: March 1, 2008 through May 31, 2008; After: March 1, 2009 through May 31, 2009. The dash “—” indicates that the information is contained in another mutually exclusive column (e.g., 2 weeks but not 8 weeks of NRT).

^3^Offered only to those ready to quit within 30 days.

^4^States who contributed seven-month follow-up data.

**Table 2:** Characteristics of tobacco users who enrolled with the 16 quitlines around the time of the April 1, 2009 federal tax increase (March 2009–May 2009) and in the same months the previous year (March 2008–May 2008) (*n* = 79,928).

	March–May 2008 *N* = 29, 674	March–May 2009 *N* = 50, 254	*P* value
Age			<0.0001
Mean (SD)	41.2 (13.7)	41.9 (13.6)	
Age	%	%	0.0005
18–24	13.6	11.5	
25–44	43.3	43.6	
45–64	38.4	39.9	
65+	4.8	5.0	
Gender	%	%	0.2822
Female	59.1	59.8	
Race/ethnicity	%	%	0.0005
White/non-Hispanic	77.5	80.1	
African American/non-Hispanic	12.1	10.4	
American Indian/non-Hispanic	5.2	4.6	
Asian/non-Hispanic	0.8	0.7	
Hispanic	4.4	4.1	
Education	%	%	0.007
≤High school	58.6	61.0	
Insurance status^1^	%	%	0.323
Uninsured	41.7	43.0	
Insured	40.5	38.3	
Medicaid	17.8	18.7	
Live/work with smoker	%	%	<0.0001
smokers at home	34.0	37.2	
smokers at work	15.4	13.1	
smokers at both	16.3	15.3	
neither	34.4	34.4	
Years of tobacco use^1^	%	%	<0.0001
0–5	3.6	5.4	
6–19	25.0	30.9	
Use Tobacco 20+ yrs	71.4	63.7	
Use after waking	%	%	0.3981
First use w/in 5 min	52.0	52.7	
Mean (s.d.) cigarettes/day			0.006
Mean (s.d.)	20.0 (12.6) *N* = 29674	20.7 (12.4) *N* = 50254	
% Mailed NRT^1^	%	%	0.176
Yes	76.3	80.9	
Tobacco use^2^	%	%	
Cigar	2.4	3.0	<0.0001
Pipe	0.3	0.5	<0.0001
Smokeless	3.9	3.6	0.017
Chronic conditions:	%	%	
Asthma	17.9	17.0	0.140
Diabetes	9.3	9.3	0.832
COPD	13.4	11.9	0.015
CAD	7.2	6.7	0.025
NONE	66.0	67.4	0.133
How heard of QL	%	%	0.0013
HCP^3^	11.4	13.1	
Family/friend	20.3	31.2	
Media	34.8	27.0	
Other	33.6	28.8	
Service Received	%	%	
% in multicall program^4^	74.7	65.7	<0.0001

^1^Some variables had missing data either because the question was not routinely asked or participants did not answer the question. Items with >10% missing data include education, insurance status, duration smoked, household smoker, and percent mailed NRT.

^2^97.3 and 97.9% (before, after) smoked cigarettes.

^3^HCP: health care provider.

^4^ *N* = 55,180 enrolled in the multicall program.

**Table 3:** Treatment outcomes at 7 months among those sampled for follow-up surveys in four states and who enrolled around the time of the federal tax increase and in the previous year.

		Registered March–May 2008	Registered March–May 2009	Unadjusted *P* values	Adjusted^1^ odds ratios (95% confidence interval)^2^
Full sample^3^: *N* = 645/1651 (39.1%)		*N* = 287/802 35.8%	*N* = 338/849 39.8%		*N* = 564/1506

% abstinent (7-day point prevalence)	Responders ITT^4^	30.7 11.0	28.7 11.4	0.59 0.77	0.95 (0.63, 1.45) 1.09 (0.77, 1.56)
% abstinent (30-day point prevalence)	Responders ITT^4^	26.8 9.6	24.9 9.9	0.57 0.84	0.96 (0.63, 1.46) 1.09 (0.76, 1.57)

In multicall program^5^: 430/1150		189/521	241/629		*N* = 417/1126

% abstinent (7-day point prevalence)	Responders ITT^4^	34.9 12.7	32.8 12.6	0.64 0.96	0.93 (0.58, 1.49)^5^ 1.16 (0.78, 1.72)^5^
% abstinent (30-day point prevalence)	Responders ITT^4^	31.8 11.5	28.6 11.0	0.48 0.77	0.91 (0.56, 1.48)^5^ 1.14 (0.76, 1.71)^5^

^1^Controlling for age, gender, race, education, chronic condition, amount smoked, how heard about quitline, and state.

^2^Before tax period is the reference group.

^3^Number of respondents/number sampled. Note that the response rate was 4% higher after tax.

^4^ITT = Intent to Treat analyses (missing outcomes = smoking).

^5^Also controlling for call program (multiple versus single), number of counseling calls completed and use of NRT.
